# The effects of white matter hyperintensities on MEG power spectra in population with mild cognitive impairment

**DOI:** 10.3389/fnhum.2023.1068216

**Published:** 2023-02-15

**Authors:** Lucia Torres-Simon, Pablo Cuesta, Alberto del Cerro-Leon, Brenda Chino, Lucia H. Orozco, Elisabeth B. Marsh, Pedro Gil, Fernando Maestu

**Affiliations:** ^1^Center of Cognitive and Computational Neuroscience, Universidad Complutense de Madrid (UCM), Madrid, Spain; ^2^Department of Experimental Psychology, Cognitive Processes and Speech Therapy, Universidad Complutense de Madrid (UCM), Madrid, Spain; ^3^Department of Radiology, Rehabilitation, and Physiotherapy, Universidad Complutense de Madrid (UCM), Madrid, Spain; ^4^Instituto de investigación Sanitaria San Carlos (IdISSC), Madrid, Spain; ^5^Institute of Neuroscience, Autonomous University of Barcelona (UAB), Barcelona, Spain; ^6^Department of Neurology, The Johns Hopkins School of Medicine, Baltimore, MD, United States; ^7^Department of Geriatric Medicine, Hospital Universitario San Carlos, Madrid, Spain

**Keywords:** cerebrovascular disease (CVD), small vessel cerebral disease, white matter hyperintensites, mild cognitive impairment, magnetoencephalography (MEG), power spectra analysis

## Abstract

Cerebrovascular disease is responsible for up to 20% of cases of dementia worldwide, but also it is a major comorbid contributor to the progression of other neurodegenerative diseases, like Alzheimer’s disease. White matter hyperintensities (WMH) are the most prevalent imaging marker in cerebrovascular disease. The presence and progression of WMH in the brain have been associated with general cognitive impairment and the risk to develop all types of dementia. The aim of this piece of work is the assessment of brain functional differences in an MCI population based on the WMH volume. One-hundred and twenty-nine individuals with mild cognitive impairment (MCI) underwent a neuropsychological evaluation, MRI assessment (T1 and Flair), and MEG recordings (5 min of eyes closed resting state). Those participants were further classified into vascular MCI (vMCI; *n* = 61, mean age 75 ± 4 years, 35 females) or non-vascular MCI (nvMCI; *n* = 56, mean age 72 ± 5 years, 36 females) according to their WMH total volume, assessed with an automatic detection toolbox, LST (SPM12). We used a completely data-driven approach to evaluate the differences in the power spectra between the groups. Interestingly, three clusters emerged: One cluster with widespread larger theta power and two clusters located in both temporal regions with smaller beta power for vMCI compared to nvMCI. Those power signatures were also associated with cognitive performance and hippocampal volume. Early identification and classification of dementia pathogenesis is a crucially important goal for the search for more effective management approaches. These findings could help to understand and try to palliate the contribution of WMH to particular symptoms in mixed dementia progress.

## Introduction

Due to high life expectancy, aged-related pathologies like dementia are rising. Alzheimer’s Disease (AD) is the most common subtype of dementia; however Vascular Dementia (VaD) or major vascular cognitive impairment (VCI) is responsible for up to 20% of cases worldwide (Catindig et al., 2012; Rizzi et al., 2014; O’Brien and Thomas, 2015; Kalaria, 2018). Besides pure VaD, cerebrovascular disease (CBVD) is also major comorbid contributor to the progression of other neurodegenerative diseases, observed to some extent in almost all patients with dementia or MCI (Khan et al., 2016), for example in 50%–90% of the patients diagnosed with AD (Santos et al., 2017), and up to 50% of dementias worldwide (Wardlaw et al., 2019). Furthermore, the presence of cerebrovascular neuropathology in post-mortem studies has been related to an increment in the risk of developing dementia even more than the evidence of AD neuropathology; but concomitant pathologies skyrocket the risk compared to people with no brain alterations, or even with evidence of exclusively Alzheimer or cerebrovascular-type lesions (Azarpazhooh et al., 2018).

CBVD involves a spectrum of medical conditions or changes in the cerebral and/or systemic vasculature with brain impact, including a huge number of pathologies and etiologies. Within these vascular pathologies, cerebral small vessel disease (CSVD) is one of the most common accompaniments of aging, not only in dementia patients but also in healthy populations. Specifically, white matter hyperintensities (WMH), seen as diffuse areas of high signal intensity (hence, “hyperintense”) on T2-weighted or fluid-attenuated inversion recovery sequences (Alber et al., 2019), are the most prevalent imaging marker on CSVD, accounting for 15% in the general population greater than 64 and more than 90% in those older than the age of 80 (Drebette and Markus, 2010; Kloppenborg et al., 2014). Although age seems to be closely related to WMH presence in the brain, the amount and progression have been associated with a higher risk of stroke, dementia, and cognitive dysfunction (Drebette and Markus, 2010; Kloppenborg et al., 2014). Specifically, WMH has been associated with small but robust effects on general cognitive impairment, attention, and executive functioning in patients with MCI and dementia (Mortamais et al., 2014; Prins and Scheltens, 2015; Arvanitakis et al., 2016; Van Den Berg et al., 2018; Lam et al., 2021). In this context, the high incidence and contribution of CBVD to cognitive impairment, added to their treatable conditions, provide special importance to the early differentiation and prevention of the pathophysiological origin of cognitive decline in the old population (Livingston et al., 2017). In addition, CBVD could help to rise more accurate prognosis and management of new therapeutic targets, with the final goal of slowing down the dementia progression.

Electrophysiologic neuroimaging techniques are of special interest for this early differentiation, as they bring useful information for assessing brain function and network dynamics revealing early changes inaccessible to standard structural imaging techniques or even to cognitive assessment. Neurophysiologic techniques, in contrast to the other functional neuroimaging techniques, provide information about brain oscillations with a wide frequency range from delta to gamma, are capable to measure the neural activation directly instead of doing it by means of indirect measures such as blood flow or metabolism and allow repeated measurements without any risk for the patients. Within the electrophysiological techniques, electroencephalography (EEG) and magnetoencephalography (MEG) are included. While EEG is less expensive and more popular in a clinical context, MEG provides a better disposition for the estimation of neural sources, a better signal to noise ratio for higher frequency bands and it is a more patient-friendly technique, and indeed it has also already come into clinical use in some hospitals for the clinical practice (Hoshi et al., 2022).

Furthermore, the study of electrophysiological spectral signatures, measured with MEG resting state recordings, has been well-established for early detection and prognosis in neurodegenerative disorders (López-Sanz et al., 2019), especially for Alzheimer’s disease (López-Sanz et al., 2018; Nakamura et al., 2018) and mild cognitive impairment (MCI; López-Sanz et al., 2016). According to the cerebrovascular factors they can also be found in studies analyzing MEG spectral features for patients with stroke (Tecchio et al., 2006; Chu et al., 2015; Kielar et al., 2016; Johnston et al., 2022), which is one of the major causes of major VCI, even using ultrasonography measures to assess carotid blood flow (Matsumoto et al., 2021). Nevertheless, to the best of our knowledge, no article has been published performing spectral analysis with MEG in older people diagnosed with mild or major VCI, and only a few can be found with EEG, as described in a recent systematic review performed by our research group (Torres-Simón et al., 2022).

In the present study, we assess MEG electrophysiological spectral signatures that differentiate between participants clinically diagnosed with MCI, according to their cognitive profile, with and without evidence of WMH of vascular origin, resulting in two groups: vascular MCI (vMCI) and non-vascular MCI (nvMCI). Based on the results previously described for EEG studies, we hypothesize that patients with vMCI will display a spectral profile compatible with more pronounced slowness of brain activity (power increase of slow bands and decrease of higher frequency bands), particularly in middle temporal regions, where WMH predominantly appears in early stages of the disease.

## Methods

The sample for the present study was recruited under the framework of two national projects (PSI2009-14415-C03-01 and PSI2012-38375-C03-01) focused on research and early detection of dementia. Data were collected between 2010 and 2014 in collaboration with three different clinical centers located in Madrid (Spain): the Neurology Department in “Hospital Universitario Clinico San Carlos,” the Center for Prevention of Cognitive Impairment, and the Seniors Center of Chamartín District. The sample consisted in 129 participants who were native Spanish speakers diagnosed with mild cognitive impairment (MCI) according to the criteria established by Petersen (2004). Patients with MCI showed cognitive impairments but did not fulfill the criteria for a dementia diagnosis. To assess general cognitive and functional status, the following set of screening questionnaires were used: The Mini Mental State Examination (MMSE; Lobo et al., 1979), the Global Deterioration Scale (Reisberg et al., 1982), the Geriatric Depression Scale–Short Form (GDS; Yesavage et al., 1982); the Functional Assessment Questionnaire (FAQ; Pfeffer et al., 1982), and the questionnaire for Instrumental Activities of Daily Living (Lawton and Brody, 1969). Participants were excluded if they had: (1) a history of psychiatric or neurological disease; and (2) psychoactive drugs consumption or chronic use of medication, such as anxiolytics. In addition, alternative etiologies of cognitive decline were ruled out, such as B12 deficiency, poorly controlled diabetes mellitus, thyroid problems, syphilis, or human immunodeficiency virus.

All participants signed an informed consent. This study was approved by the “Hospital Universitario Clinico San Carlos” ethics committee and was performed in accordance with approved guidelines and regulations.

### Neuropsychological assessment

All patients underwent an additional neuropsychological assessment to generate detailed cognitive profiles including Direct and Inverse Digit Span Test (Wechsler Memory Scale, WMS-III; Wechsler, 1997), Immediate and Delayed Recall (WMS-III; Wechsler, 1997), and Phonemic and Semantic Fluency (Controlled Oral Word Association Test, COWAT; Benton and Hamsher, 1989).

### Magnetic resonance recordings and analysis

T1-weighted and T2-weighted 3D FLAIR MRI were obtained using a General Electric 1.5 Tesla magnetic resonance scanner, with a high-resolution antenna and a homogenization PURE filter (Fast Spoiled Gradient Echo sequence). The following parameters were used for T1-weighted imaging: repetition time (TR) = 11.2 ms, echo time (TE) = 4.2 ms, inversion time (TI) = 450 ms, Field Of View (FOV) = 25 cm, flip angle (FA) = 12°, 252 coronal slices (in-plain resolution: 256 × 256), voxel size: 0.98 × 0.98 × 1 mm^3^ and acquisition time ≃8:00 min. T2-weighted 3D FLAIR images were obtained with the following specifications: TR = 7,000 ms, TE = 101 ms, TI = 2,112 ms, FOV = 24 cm, 252 coronal slices (in-plain resolution: 256 × 112), voxel size: 0.94 × 0.94 × 1.6 mm^3^ and acquisition time ≃4:57 min.

WMH segmentation (T1 and Flair) and lesion volume calculation were performed using an automatic computerized toolbox in SPM12, called Lesion Segmentation Tool (LST). We used the initial κ threshold with default value of 0.3 as recommended by the developers (Schmidt et al., 2012), and subsequently the probabilistic threshold of 0.5, as it was the most accurate compared with the clinician’s manual segmentations.

### WMH volume cut point

As explained before in the introduction, a minimum load WMH is present in practically every individual above 60 years old. Nevertheless, the amount and progression of this vascular marker has been closely related with a higher risk of stroke and dementia and also with cognitive dysfunction. In order to establish a possible threshold from which WMH volume in the brain could have clinical implications we have calculated a specificity-based cut point for the WMH damage quantity (Jack et al., 2017). For this purpose, we used the WMH segmentations of a preexisting dataset of 346 cognitively intact (CI) older individuals without evidence of vascular disease, according to the radiological report. This cut point corresponded to the 95th percentile of the WMH damage quantity distribution among CI individuals aged 50–86 years (average 67 ± 8). The obtained cut point corresponded to a WMH volume of 3,448 mm^3^.

### MEG data acquisition

Five minutes of resting-state (closed-opened eyes) were recorded with a Vectorview system (Elekta Neuromag) at the Center for Biomedical Technology (Madrid, Spain). All participants sat comfortably in a chair with their eyes closed. The arousal level of each subject was monitored with a video camera and checked *via* a conversation immediately following the measurement session. MEG data were collected at a sampling frequency of 1,000 Hz and online band-pass filtered between 0.1 and 330 Hz. Each subject’s head shape was defined relative to three anatomical locations (nasion and bilateral preauricular points) using a 3D digitizer (Fastrak, Polhemus, VT, USA) and the head motion was tracked through four head-position indicator (HPI) coils attached to the scalp. These HPI coils continuously monitored the participants’ head movements, while eye movements were monitored by a vertical electrooculogram assembly (EOG) composed of a pair of bipolar electrodes. Raw recording data were first introduced to Maxfilter software (v 2.2, correlation threshold = 0.9, time window = 10 s) to remove external noise using the temporal extension of the signal space separation method with movement compensation (Taulu and Simola, 2006). Then, magnetometers data (Garcés et al., 2017) were automatically examined to detect ocular, muscle, and jump artifacts using Fieldtrip software (Oostenveld et al., 2011), which were visually confirmed by an MEG expert (initials). Twelve participants were dismissed due to bad quality MEG data, resulting in a sample of 117 participants. The remaining artifact-free data was sectioned into 4-s segments. Independent component analysis-based procedure (ICA) was applied to remove heart magnetic field artifacts and EOG components. Only those recordings with at least 20 clean segments (80 s of brain activity) were utilized in subsequent analyses.

MEG clean time series were band-pass filtered (2 s padding) between 2 and 45 Hz. Source reconstruction was carried out using a regular grid of 1 cm spacing in the Montreal Neurological Institute (MNI) template. The resulting model comprised 2,459 sources homogeneously distributed across the brain. This model was linearly transformed to each subject’s space. The leadfield was calculated using a single shell model (Nolte, 2003). Source time series were reconstructed using a Linearly Constrained Minimum Variance beamformer (Van Veen et al., 1997). The power spectrum of each grid’s node was computed by means of a multitaper method (mtmfft) with discrete prolate spheroidal sequences (dpss) as windowing function and 1 Hz smoothing. For each node, relative power was calculated by normalizing by total power over the 2- to 30-Hz range. The source template with 2,459 nodes in a 10 mm spacing grid was segmented into 78 regions of the Automated Anatomical Labeling atlas (AAL, Tzourio-Mazoyer et al., 2002), excluding the cerebellum, basal ganglia, thalamus, and olfactory cortices. Those 78 regions of interest included 1,202 of the original 2,459 nodes. Trials were averaged across participants ending up with a source-reconstructed power matrix of 1,202 nodes × 113 frequency steps × 117 participants.

### Statistical procedure

The assessment of significance between group power differences was addressed by relying on the cluster-based permutation test (CBPT; Maris and Oostenveld, 2007; Zalesky et al., 2010). Power differences were tested between groups for each pair of nodes using an ANCOVA test while adjusting for the effects of age and total white matter volume. The resulting matrix of F-statistics (with the same dimension of original power matrix) was binarized by thresholding the matrix using a critical value computed with a *p*-value of 0.005. This binary matrix was split into two matrices attending for the sign of the differences between groups. Clusters were built by grouping spatially adjacent nodes that systematically showed significant between-groups differences. All nodes within a cluster must exhibit the same sign of between-groups differences, indicating that the cluster was likely a functional unit. Only clusters involving at least 1% of the nodes (i.e., a minimum of 12 nodes) were considered. Cluster-mass statistics were assessed through the sum of all F-values across all nodes. To control for multiple comparisons, the entire analysis pipeline was repeated 10,000 times after shuffling the original group’s labels. At each repetition, the maximum statistic of the surrogate clusters was kept, creating a maximal null distribution that would ensure control of the family-wise error rate (FWER) at the cluster level. Cluster-mass statistics on each cluster in the original data set was compared using the same measure in the randomized data. The CBPT *p*-value represented the proportion of the permutation distribution with cluster-mass statistic values greater or equal to the cluster-mass statistic value of the original data. Only those clusters that survived the CBPT at *p* < 0.05 or below were considered for the subsequent analyses as potential “MEG markers.” As descriptive values for each significant cluster, we computed the average power (across all nodes that belong to the cluster). These values were used as MEG marker values for the subsequent Spearman correlation analysis with measures of cognitive performance and structural integrity. In addition, we computed between groups statistics for these averaged power values using ANCOVA with age and total white matter volume as covariates. Statistical analyses were carried out using Matlab R2021b (Mathworks Inc).

### Data availability

The data that support the findings of this study are available from the corresponding author, upon request. All the algorithms used in the present article are reported in the “Methods” section.

## Results

### Segregation of patients with MCI based on their WMH volume

The aim of this piece of work is the assessment of brain functional differences in an MCI population based on the WMH volume, and their possible correlation with cognitive performance. The original 117 patients with MCI were divided into two groups (VMCI vs. nVMCI) using their WMH volumes and the 3,448 mm^3^ cut point. Sixty-one patients diagnosed with MCI showed WMH volumes above the cut point (hence vMCI group) whereas 56 had WMH volumes below the cut point (nvMCI group). Both groups were compared to assess possible differences in all demographic scores displayed in Table 1. Patients with vMCI showed significantly higher age (*p* < 0.001), Total gray matter volume (GM volume; *p* = 0.014) and WMH volume (*p* ⋘ 0.001). No significant differences between groups arise when cognitive performance and hippocampal volumes were assessed. This last result is important since cognition and hippocampal atrophy are common markers of dementia status. Descriptive data and statistical scores are depicted in Table 1.

**Table 1:** Participants’ demographics.

	**vMCI (61)**	**nvMCI (56)**	***p*-value**	***F* value**	***η^2^***
Sex (females)	35	36	0.46		
Age	75.26 ± 4.19	72.13 ± 5.11	<0.001*	13.28*	0.104
Education (years)	9.08 ± 4.67	8.44 ± 4.14	0.21	1.60	0.026
MMSE	26.15 ± 2.68	27.09 ± 2.48	0.92	0.01	<0.001
Immediate Recall	15.13 ± 8.07	16.70 ± 8.92	0.89	0.02	<0.001
Delayed Recall	5.23 ± 6.41	6.42 ± 6.80	0.87	0.03	<0.001
Dígit Spam Forward	6.95 ± 1.74	6.54 ± 1.66	0.09	3.00	0.048
Dígit Spam Backward	4.03 ± 1.37	4.20 ± 1.54	0.55	0.36	0.006
Phonemic Fluency	8.28 ± 4.25	8.40 ± 3.77	0.54	0.38	0.006
Semantic Fluency	11.77 ± 2.94	12.48 ± 3.96	0.55	0.37	0.006
GDS	3.89 ± 2.89	4.45 ± 3.50	0.12	2.50	0.041
Apoe-ε4	27	20	0.45		
WMH volume	(10 ± 6) · 10^3^	(1 ± 1) · 10^3^	⋘0.001	60.17	0.505
WMH number of lesions	23 ± 13	12 ± 8	0.06	3.70	0.059
GM volume	(531 ± 51) · 10^3^	(527 ± 48) · 10^3^	0.014	6.36	0.097
WM volume	(389 ± 50) · 10^3^	(395 ± 57) · 10^3^	0.53*	0.40*	0.003
LHV	0.0022 ± 0.0003	0.0023 ± 0.0004	0.87	0.03	<0.001
RHV	0.0022 ± 0.0003	0.0024 ± 0.0004	0.80	0.07	0.001

Values are presented as mean ± SD. MMSE, mini-mental state examination; GDS, geriatric depression scale; WMH volume, white matter hyperintensities total volume; WMH number of lesions, white matter hyperintensities number of lesions; GM volume, total gray matter volume (mm^3^); WM volume, total white matter volume (mm^3^); L/RHV, left/right hippocampal volume. Medial temporal lobe volumes were normalized with respect to the overall intracranial volume to account for differences in head volume among participants. *p*-values correspond with ANCOVA with age and WM volume as covariates, *t*-test (for age and WM volume, marked with *), and Fisher (sex and ApoE-ε4) tests- *F* values account for ANCOVA analysis. For age and WM volume the values correspond to t values. η^2^ accounts for partial-Eta squared for all comparisons but for the case of age and WM volume l where the values correspond to η^2^ values.

### Electrophysiological power differences between groups

The power spectra were used to determine the brain regions and frequency ranges with a significant difference between vMCI and nvMCI groups. The results showed three significant clusters.

**Figure 1:**
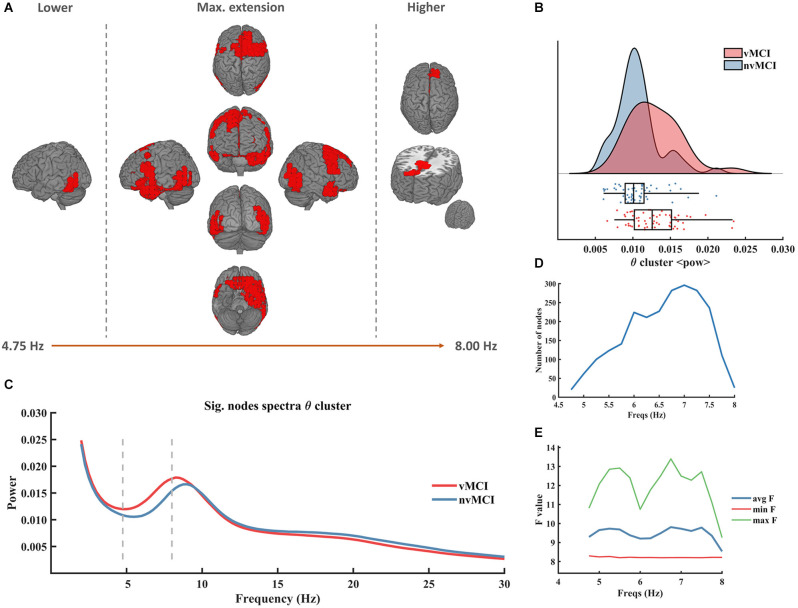
Significant result found within the theta frequency range. **(A)** Description of the regions involved in the θ cluster. The cluster was found to be significant between 4.75 Hz and 8.00 Hz. In the figure can be seen the initial, final, and maximum extensions of the cluster. **(B)** Violin plots and boxplots graphics describing the individual values for the average power of the cluster in the significant frequency range. **(C)** Representation of the average spectral power across all significant nodes. The significant frequency region is marked with dashes lines. **(D)** Number of grid nodes that are part of the cluster at each frequency step (maximum extension was found at 7 Hz). **(E)** Minimum, maximum, and average *F* values across all nodes contained within the cluster at each frequency step.

The first significant result (named θ cluster, CBPT *p* value = 0.036) emerged as a widespread cluster (see Figure 1A), whose brain oscillatory activity in the frequency range [4.75–8.00 Hz] (Figure 1C) differed between groups. The power of this cluster, that fell within the classical theta band range, was enhanced in the vMCI group when compared to the nvMCI group (see Figure 1B). The cluster size ranged between 20 nodes up to 300 nodes reaching the maximum size at the frequency of 7 Hz (see Figure 1D). The average F value, across all nodes of the cluster, is depicted in Figure 1E. A detailed description of the ROIs involved in the θ cluster can be found in Supplementary Table 1.

**Figure 2:**
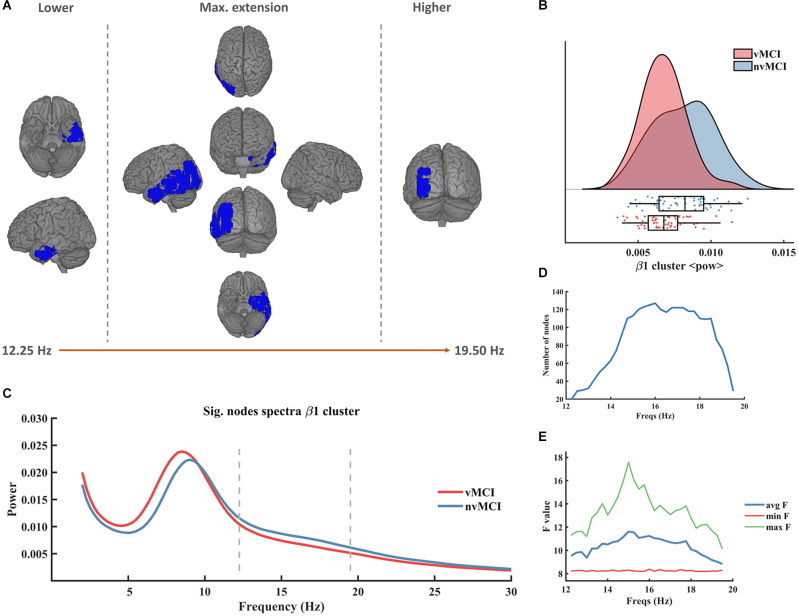
Significant result found within the beta frequency range in the left temporo-parietal region. **(A)** Description of the regions involved in the β1 cluster. The cluster was found to be significant between 12.25 Hz and 19.50 Hz. In the figure can be seen the initial, final, and maximum extensions of the cluster. **(B)** Violin plots and boxplots graphics describing the individual values for the average power of the cluster in the significant frequency range. **(C)** Representation of the average spectral power across all significant nodes. The significant frequency region is marked with dashes lines. **(D)** Number of grid nodes that are part of the cluster at each frequency step (maximum extension was found at 16 Hz). **(E)** Minimum, maximum, and average *F* values across all nodes contained within the cluster at each frequency step.

**Figure 3:**
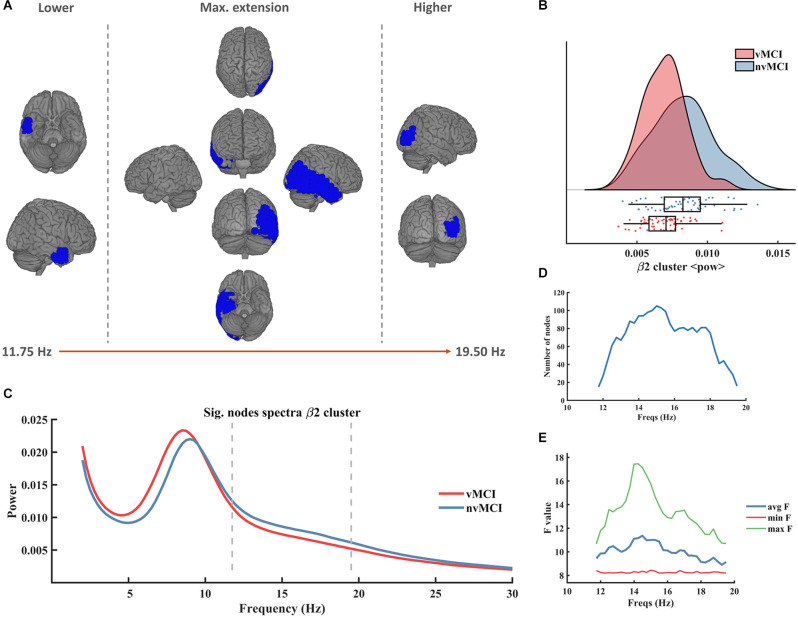
Significant result found within the beta frequency range in the right temporo-parietal region. **(A)** Description of the regions involved in the β2 cluster. The cluster was found to be significant between 11.75 Hz and 19.50 Hz. In the figure can be seen the initial, final, and maximum extensions of the cluster. **(B)** Violin plots and boxplots graphics describing the individual values for the average power of the cluster in the significant frequency range. **(C)** Representation of the average spectral power across all significant nodes. The significant frequency region is marked with dashes lines. **(D)** Number of grid nodes that are part of the cluster at each frequency step (maximum extension was found at 15 Hz). **(E)** Minimum, maximum, and average *F* values across all nodes contained within the cluster at each frequency step.

Besides from the θ cluster, two significant results showing a diminished power in the vMCI group when compared to the individuals included in nvMCI, were found within the beta frequency band (Figures 2B and 3B).

The second beta cluster (henceforth called β2, CBPT *p* value = 0.0401) involved right temporo-parietal regions of the brain (see Figure 3A). The β2 cluster contralaterally mirrored the result of the β1 cluster since it showed a decreased beta power in the patients with vMCI when compared to patients without vascular damage in the right hemisphere of the brain (Figure 3B). It was defined in the frequency range [11.75 Hz to 19.50 Hz] (Figure 3C). The size of the cluster fluctuated between 15 and 105 nodes, peaking at 15 Hz (Figure 3D). The maximum average *F* value, across all nodes of the cluster, was found at 14 Hz (Figure 3E). A detailed description of the ROIs involved in the β2 cluster can be found in Supplementary Table 3.

The first beta cluster (henceforth called β1, CBPT *p* value = 0.0336) involved left temporo-parietal regions of the brain (see Figure 2A). It emerged in the frequency range [12.25 Hz to 19.50 Hz] (Figure 2C). The size of the cluster ranged between 20 and 127 nodes, peaking at 16 Hz (Figure 2D). The maximum average F value, across all nodes of the cluster, was found at 15 Hz (Figure 2E). A detailed description of the ROIs involved in the β1 cluster can be found in Supplementary Table 2.

### Correlations between electrophysiological markers and brain health

In order to help the interpretation of the results, we carried out Spearman correlation test between the average power of each cluster and scores of brain health (neurophysiological assessment and structural quantitative scores associated with gray matter atrophy). The significant results are depicted in Table 2, and the scores included in the analysis are those described in Table 1.

**Table 2:** Significant correlation between power markers and brain health.

	**θ cluster**		**β1 cluster**		**β2 cluster**	
	**rho**	***p* val**	**rho**	***p* val**	**rho**	***p* val**
MMSE	−0.306	0.001	0.269	0.004	0.288	0.002
Dígit Spam Backward					0.184	0.050
Immediate Recall			0.207	0.027		
Delayed Recall	−0.314	0.001	0.303	0.001	0.250	0.008
Semantic Fluency			0.188	0.049		
LHV	−0.261	0.005	0.225	0.016	0.210	0.024
RHV	−0.282	0.002	0.246	0.008	0.281	0.002

Spearman correlation analyses between the average power of each corresponding cluster and brain health in the whole sample. All correlations were computed with age and total white matter volume as covariates. MMSE, mini-mental state examination; L/RHV, left/right hippocampal volume normalized by total intracranial volume. Phonological fluency was not included in the table because it did not significantly correlate with any MEG marker.

As it can be seen in Table 2, all three power markers correlated significantly with neuropsychological performance and hippocampal integrity. Importantly, whilst the average power of the θ cluster correlated negatively with cognitive and structural integrities, both β clusters showed a positive correlation. This indicates that brain health seems to be associated with decreased θ power and increased β power. This association emerged clearly when assessing correlations between all power markers and hippocampal volumes, delayed recall and MMSE performance. These results emphasize the relevance of the MEG markers for early differentiation since both MCI groups did not show statistical differences in cognition nor in hippocampal volumes.

## Discussion

In this study, we compared the spectral profiles during resting state, assessed with MEG, for two MCI populations differentiated according to their level of cerebrovascular damage, described as the total volume of white matter hyperintensities in the brain. As we hypothesized, our results demonstrated the emergence of increased power for slow bands, specifically a widespread theta band (5–9 Hz), and smaller power was seen for high-frequency bands, in particular in the beta band (12–20 Hz) in bilateral temporal regions. Importantly these power spectral changes were independent of age, gender, genetic predisposition, or cognitive impairment, as they were controlled for our analysis.

To the best of our knowledge, this is the first study using MEG spectral analysis to compare MCI populations with and without cerebrovascular damage, specifically assessing the presence of WMH. The lack of literature regarding electrophysiological spectral patterns measured with MEG related to VCI makes it impossible to directly compare our results with previous literature, but we can confirm that our findings are in line with those described in EEG studies (Torres-Simón et al., 2022). It is worth mentioning that in many of these EEG studies, the inclusion criteria for the VCI population are vague or broad. In fact, in most cases, it is not possible to differentiate between VCI subtypes or to assess the degree of comorbidity in MCI or dementia patients with mixed pathology. Few articles described clear inclusion and exclusion criteria regarding MRI standards, specifically for WMH. Additionally, in most of these studies, structural vascular damage or white matter damage of vascular origin was assessed using internationally validated clinical visual scales. These scales are the most commonly used method to detect and evaluate the severity of WMH, being the Fazekas scale (Fazekas et al., 1987), and the age-related white matter changes scale (ARWMC; Wahlund et al., 2001) the most recommended visual rating (Wahlund et al., 2017). Nevertheless, these scales do not report true quantitative information (i.e., lesion volume), which makes the evaluation of the progression difficult when the changes are not abrupt. In this context, a clinician’s manual segmentation is the most accurate method to measure lesion volume in white matter and it is considered as the gold standard (Commowick et al., 2018). Notwithstanding, segmenting the WMH manually is extremely time-consuming, and it comprises high inter-rater variability making its application in clinical and scientific practice unfeasible. For this reason, automatic segmentation tools are increasing the presence in this research field and allow for direct comparison of WMH between groups. Due to its great balance between accuracy and time dedication, we decided to include this automatic analysis to classify as objectively as possible our patients in the two experimental groups according to their level of cerebrovascular damage for later comparison.

### Increment in theta power

An increase in theta power during the resting state for vMCI compared to nvMCI has been broadly reported. Previous literature reports that the presence and severity of cerebrovascular damage is related to a higher increment of power in the theta band. Specifically, it has been reported that patients with mild cognitive impairment and evidence of vascular damage experienced an augmentation in theta band power compared to healthy age-matched controls (Gawel et al., 2007; van Straaten et al., 2012); but interestingly, this increment was even greater for patients with vMCI than for patients with nvMCI (Babiloni et al., 2004; Moretti et al., 2004, 2007; Gawel et al., 2009; Sheorajpanday et al., 2013; Quandt et al., 2020). Furthermore, this theta band power increment has been closely linked to general cognitive impairment, assessed with MMSE (Moretti et al., 2007). We are pleased to confirm this correlation in our sample. Additionally, we found a similar negative correlation between the power of the theta cluster and delayed recall scores. This result helps to confirm that the increased power found could be considered the reflection in the brain function of poorer cognitive functioning, although non-significant, derived from the cerebrovascular damage in our patients, which could help to the prognosis of worse memory decline in the future.

While the association between increased theta power, age, and cognitive impairment (Schomer and Lopes da Silva, 2012) is well established, our data suggest that evidence of cerebrovascular pathologies (i.e., WMH) exacerbates this relationship. The sharper increment in slow frequency bands found for vMCI compared to nvMCI with similar severity of cognitive impairment could be explained by pathological changes in the brain driven by WMH. The referred hyperintense lesions are predominantly located in the deep white matter of the brain and are presumably related to small vessel disease, in which little ischemic events increase white matter injuries triggering axonal damage and even diffuse demyelination (Jang et al., 2017; Venkat et al., 2017; Kalaria, 2018); increase in blood-brain barrier permeability, contributing to neurodegeneration, apoptosis, and functional disruption (Farrall and Wardlaw, 2009; Schreiber et al., 2013); dysregulation of neurotransmitter systems, such as the cholinergic system (Caruso et al., 2019); and alterations of cerebral blood flow and chronic hypoperfusion (Yang et al., 2002; Tak et al., 2011). Brain white matter tracks are able to connect cortical and subcortical gray matter across a relatively large distance, as temporal, and frontal lobes whose interaction is tightly related to theta wave generation (Anderson et al., 2010). Consequently, an increase in widespread theta could be explained as a cortical oscillatory activity reflection of deep white matter injury. Going a step further in this hypothesis, in the present study, we found that the theta band cluster that emerged was negatively associated with bilateral hippocampal volume. The higher WMH total volume, the larger the theta band power in the cluster and the lower the hippocampal volumes. This correlation seems especially important to explain the possible association between cognitive impairments with periventricular WMH, which has been previously hypothesized to be reflecting the disruption of cholinergic projections from the basal forebrain to the cortex (Alber et al., 2019).

### Decrement in beta power

The positive relationship between a reduction in beta band power in resting state and general cognitive functioning (MMSE, delayed and immediate memory performance, semantic fluency, and attentional spam) has been previously reported. Specifically, prior studies show smaller beta (β) band power in VCI patients compared with HC, once again associated with greater evidence of cognitive symptomatology (van Straaten et al., 2012; Al-Qazzaz et al., 2017). Unfortunately, there were no comparisons made to patients with nvMCI or AD. Additionally, previous studies obtained differences in alpha band for vMCI compared with HC associated with increased severity of cerebrovascular damage and cognitive impairment. They observed decrement in the low-alpha (Babiloni et al., 2004; Moretti et al., 2007; Sheorajpanday et al., 2013; Wu et al., 2014) and high-alpha (Moretti et al., 2004; Al-Qazzaz et al., 2017) power and a slower peak alpha frequency (Moretti et al., 2004; Neto et al., 2015). All these results are in line with our main hypothesis that stated that a more pronounced slowing of spectral brain activity could be related to the presence and progression of brain damage of vascular origin. The heterogeneity in the alpha band definition and naming could be distorting these results, meaning that similar underlying electrophysiological phenomena have been described as different findings.

In contrast to the widespread presentation of the theta power alteration, the decrement in beta is explicitly localized in bilateral temporal regions. We are pleased to see that our findings go in line with the well-stated idea that slower oscillatory powers are primarily produced by the thalamo-cortical network, while faster oscillatory powers depend on local activities (Klimesch, 1999) Additionally, beta band functioning alteration has been widely reported in studies focused on patients with ischemic strokes (Kulasingham et al., 2022; Pusil et al., 2022). Interestingly, the clusters that emerged in the present study for lower beta band power overlap the deepest brain regions surrounding periventricular areas where WMH most predominantly appears and is strongly related to the positive relationship that we found with both beta temporal clusters with right and left hippocampal volumes. As previously explained in the introduction, WMH is the neuroimaging evidence of the small and recurrent ischemic events underlying the cerebral small vessels disease (CSVD). In this context, our finding related to local beta power reduction hypothetically could explain some kind of relation between beta band local functioning and brain ischemic events, but this is something that needs further investigation.

## Limitations

The lack of previous literature assessing those with vMCI using MEG is one of the main concerns of the present study as it does not allow us to directly compare our results. This lack of replication should be considered for results generalization, but it also entails the beginning of a new and unexplored research field.

Although all the patients included in the sample met the MCI diagnosis criteria (Petersen, 2004), the lack of neuropathological markers of AD (i.e., CSF/PET amyloid or tau markers) potentially increases the heterogeneity of the MCI population and could diminish the degree of association between VCI and AD that we can state with our data. However, significant differences were found despite this limitation.

Finally, recent meta-analysis found that WMH volume estimation was not comparable between studies due to the lack of standardization in the definition of WMH and the high technical variability in assessment (Melazzini et al., 2021). Despite the lack of evidence for WMH volume references intervals, the literature strongly supports the role of WMH as a biomarker of longstanding cerebrovascular disease, and its implication in the pathophysiology of stroke and cognitive impairment or dementia (Chutinet and Rost, 2014). With the intention of advancing in the search for possible thresholds for this imaging marker we have calculated and detailed reported our findings and procedure, based on a referenced method for defining biomarker cut points for brain aging (Jack et al., 2017), to facilitate further replication.

## Conclusions

The lack of literature on electrophysiological spectral patterns related to patients with mild (referred to in this study as vMCI) or major VCI with MEG, and the lack of consensus and replication on EEG results evidence that electrophysiology signatures are not ready yet to be included in the diagnosis criteria for cognitive impairment of vascular origin because they are not robust, repeatable, and reliable enough to be used as clinical biomarkers. MEG quantitative analysis has been previously stated as a precise, non-invasive tool with high temporal resolution that is able to reflect changes in the bioelectrical activity of the brain. This brings us the opportunity to study brain functioning disruption due to changes in synaptic potentials produced by vasculature alterations before structural changes and/or cognitive decline are evidenced. Nevertheless, there is a critical need to accurately classify VCI in future electrophysiological research in order to facilitate scientific results sharing and aggregation. CBVD underlying cognitive impairment of vascular origin involves a variety of medical conditions, pathologies, and etiologies. In this sense, trying to look for biomarkers generalizable to all of them is practically impossible. In this context, we encourage a reductionist approach oriented to find specific electrophysiological signatures related to this specific cerebrovascular damage (i.e., WMH) reducing the incidence of other possible pathological variables, trying to establish a strong and accurate baseline for VCI research. This approach could help to understand and try to palliate particular symptoms in mixed dementias, where WMH damage is a comorbid contributor to the progression of other neurodegenerative diseases. The present study establishes a baseline for MEG future research in the VCI field, showing general electrophysiological spectral patterns, registered with MEG. These results demonstrate the utility of MEG signal analysis to augment structural imaging studies in the differentiation of MCI subtypes and to understand the effect of CBVD in brain function and cognition.

## Data availability statement

The raw data supporting the conclusions of this article will be made available by the authors, without undue reservation.

## Ethics statement

The studies involving human participants were reviewed and approved and this study was approved by the “Hospital Universitario Clinico San Carlos” ethics committee and was performed in accordance with approved guidelines and regulations. The patients/participants provided their written informed consent to participate in this study.

## Author contributions

LT-S: conceptualization, methodology, formal analysis, investigation, writing—original draft, writing—review and editing, visualization, project administration, and funding acquisition. PC: conceptualization, methodology, formal analysis, investigation, writing—original draft, writing—review and editing, visualization and supervision. AC-L: methodology, formal analysis, investigation, and writing—original draft. BC: writing—review and editing, supervision and funding acquisition. LO: investigation, and writing—original draft. EM: writing—review and editing, supervision, and validation. PG: writing—review and editing, supervision, validation and funding acquisition. FM: writing—review and editing, resources, supervision, validation and funding acquisition. All authors contributed to the article and approved the submitted version.
