# Supplementary material for: The effects of white matter hyperintensities on MEG power spectra in population with mild cognitive impairment
Source: Front Hum Neurosci. 2023 Feb 15;17:1068216. doi: 10.3389/fnhum.2023.1068216 (PMC9977191; doi:10.3389/fnhum.2023.1068216)
Supplement: Supplementary file 1 [file Table_1.docx]

Supplementary Materials

| **Supplementary Materials Table 1**: Cluster θ description | | | |
| --- | --- | --- | --- |
| **ROI** | **% ROI** | **% Clust** | **Avg F value** |
| lMTG | 20,45 | 3,04 | 11,06 |
| lTPsup | 100,00 | 3,38 | 10,77 |
| lInsula | 100,00 | 4,73 | 10,58 |
| lIFGo | 100,00 | 2,36 | 10,56 |
| lTPmid | 85,71 | 2,03 | 10,39 |
| lAmyg | 100,00 | 0,68 | 10,30 |
| rMotor | 71,43 | 3,38 | 10,13 |
| lITG | 50,00 | 4,05 | 10,06 |
| rMFG | 32,50 | 4,39 | 10,01 |
| rTPmid | 40,00 | 1,35 | 9,96 |
| rMTG | 10,81 | 1,35 | 9,95 |
| lACC | 100,00 | 6,42 | 9,92 |
| rPreCG | 33,33 | 3,04 | 9,91 |
| rMCC | 73,33 | 3,72 | 9,79 |
| lIFGo | 83,33 | 3,38 | 9,77 |
| lSTG | 40,00 | 2,70 | 9,74 |
| lHip | 80,00 | 1,35 | 9,72 |
| lRO | 60,00 | 1,01 | 9,63 |
| rSFG | 61,29 | 6,42 | 9,62 |
| rIFGor | 61,54 | 2,70 | 9,62 |
| rSFGmo | 40,00 | 0,68 | 9,61 |
| lSFGmo | 50,00 | 1,01 | 9,58 |
| lRectus | 75,00 | 2,03 | 9,54 |
| lMFGo | 42,86 | 1,01 | 9,51 |
| rACC | 100,00 | 1,69 | 9,50 |
| lMCC | 50,00 | 2,70 | 9,44 |
| rTPsup | 62,50 | 1,69 | 9,41 |
| lMotor | 66,67 | 5,41 | 9,40 |
| lSFG | 14,81 | 1,35 | 9,31 |
| rSFGm | 40,00 | 2,03 | 9,23 |
| lPosG | 2,94 | 0,34 | 9,22 |
| rIFGt | 10,53 | 0,68 | 9,18 |
| lIFGt | 57,14 | 4,05 | 9,15 |
| lParahip | 37,50 | 1,01 | 9,14 |
| lFusiG | 33,33 | 1,69 | 9,12 |
| rInsula | 35,71 | 1,69 | 9,05 |
| lPreCG | 21,88 | 2,36 | 9,00 |
| rSFo | 33,33 | 0,34 | 8,79 |
| rIFGo | 25,00 | 1,01 | 8,73 |
| rRectus | 75,00 | 1,01 | 8,64 |
| lMFG | 14,71 | 1,69 | 8,58 |
| lSFGm | 23,53 | 2,70 | 8,53 |
| rMFGo | 14,29 | 0,34 | 8,44 |
| **% ROI** = percentage of the ROI within the cluster. **% Clus** = percentage of the cluster within the ROI. **Avg F value** = average F value obtained for the comparison between group across all nodes involved within the corresponding ROI. ROIs were ordered based on their cluster size percentage. **r/l**=right/left. **ACC** = Cingulate gyrus, Anterior part; **Amyg** = Amygdala; **Calc** = Calcarine fissure and surrounding cortex; **FusiG** = Fusiform gyrus; **Hip** = Hippocampus; **IFGo** = Inferior Frontal gyrus, Orbital; **IFGop** = Inferior Frontal gyrus, Opercular; **IFGt** = Inferior Frontal gyrus, Triangular; **IOccL** = Inferior Occipital lobe; **ITG** = Inferior Temporal gyrus; **Lingual** = Lingual gyrus; **MCC** = Cingulate gyrus, Middle part; **MFG** = Middle Frontal gyrus; **MFGo** = Middle Frontal gyrus, Orbital; **Motor** = Supplementary Motor area; **MTG** = Middle temporal gyrus; **Parahip** = Parahippocampus; **ParaL** = Paracentral lobule; **PosG** = Postcentral gyrus; **PreCG** = Precentral gyrus; **Rectus** = gyrus rectus; **RO** = Rolandic operculum; **SFG** = Superior Frontal gyrus; **SFGm** = Superior Frontal gyrus, Medial; **SFGmo** = Superior Frontal gyrus, Medial Orbital; **SMG** = Supramarginal gyrus; **STG** = Superior Temporal gyrus; **TPmid** = Temporal pole, Middle temporal gyrus; **TPsup** = Temporal pole, Superior Temporal gyrus. | | | |

| **Supplementary Materials Table 2**: Cluster β1 description | | | |
| --- | --- | --- | --- |
| **ROI** | **% ROI** | **% Clust** | **Avg F value** |
| lHip | 100 | 3,937 | 12,84 |
| lITG | 95,8333 | 18,1102 | 12,12 |
| lFusiG | 100 | 11,811 | 12,04 |
| lParahip | 87,5 | 5,5118 | 11,91 |
| lPrecu | 7,1429 | 1,5748 | 11,55 |
| lMTG | 84,0909 | 29,1339 | 11,06 |
| lIOccL | 50 | 3,1496 | 10,78 |
| lMOccL | 51,7241 | 11,811 | 10,74 |
| lAng | 22,2222 | 1,5748 | 10,58 |
| lAmyg | 100 | 1,5748 | 10,00 |
| lLingual | 35,7143 | 3,937 | 9,83 |
| lCalc | 10 | 1,5748 | 9,78 |
| lSTG | 30 | 4,7244 | 9,57 |
| lTPmid | 28,5714 | 1,5748 | 8,63 |
| **% ROI** = percentage of the ROI within the cluster. **% Clus** = percentage of the cluster within the ROI. **Avg F value** = average F value obtained for the comparison between group across all nodes involved within the corresponding ROI. ROIs were ordered based on their cluster size percentage. **r/l**=right/left. ; **Amyg** = Amygdala; **Ang** = angular gyrus; **Calc** = Calcarine fissure and surrounding cortex; **FusiG** = Fusiform gyrus; **Hip** = Hippocampus; **IOccL** = Inferior Occipital lobe; **ITG** = Inferior Temporal gyrus; **Lingual** = Lingual gyrus; **MOccL** = middle occipital lobe; **MTG** = Middle temporal gyrus; **Parahip** = Parahippocampus; **Precu** = precuneus; **STG** = Superior Temporal gyrus; **TPmid** = Temporal pole, Middle temporal gyrus. | | | |

| **Supplementary Materials Table 3**: Cluster β2 description | | | |
| --- | --- | --- | --- |
| **ROI** | **% ROI** | **% Clust** | **Avg F value** |
| rMTG | 83,7838 | 29,5238 | 11,86 |
| rITG | 88,4615 | 21,9048 | 11,71 |
| rFusiG | 78,9474 | 14,2857 | 10,80 |
| rHip | 85,7143 | 5,7143 | 10,45 |
| rSTG | 62,963 | 16,1905 | 10,09 |
| rMOccL | 11,7647 | 1,9048 | 9,87 |
| rInsula | 7,1429 | 0,9524 | 9,74 |
| rParahip | 50 | 4,7619 | 9,67 |
| rAng | 16,6667 | 2,8571 | 9,03 |
| rIOccl | 40 | 1,9048 | 8,87 |
| **% ROI** = percentage of the ROI within the cluster. **% Clus** = percentage of the cluster within the ROI. **Avg F value** = average F value obtained for the comparison between group across all nodes involved within the corresponding ROI. ROIs were ordered based on their cluster size percentage. **r/l**=right/left. ; **Ang** = angular gyrus; **FusiG** = Fusiform gyrus; **Hip** = Hippocampus; **IOccL** = Inferior Occipital lobe; **ITG** = Inferior Temporal gyrus; **MOccL** = middle occipital lobe; **MTG** = Middle temporal gyrus; **Parahip** = Parahippocampus; **STG** = Superior Temporal gyrus; | | | |
